# The expression of telomere-related proteins and DNA damage response and their association with telomere length in colorectal cancer in Saudi patients

**DOI:** 10.1371/journal.pone.0197154

**Published:** 2018-06-05

**Authors:** Ftoon Aljarbou, Nourah Almousa, Mohammad Bazzi, Sooad Aldaihan, Mohammed Alanazi, Othman Alharbi, Majid Almadi, Abdulrahman M. Aljebreen, Nahla Ali Azzam, Maha Arafa, Abeer Aldbass, Jilani Shaik, Shaheerah Alasirri, Arjumand Warsy, Abdullah Alamri, Narasimha Reddy Parine, Ghadah Alamro

**Affiliations:** 1 Genome Research Chair, Department of Biochemistry, College of Science, King Saud University, Riyadh, Saudi Arabia; 2 Division of Gastroenterology, King Khalid University Hospital, King Saud University, Riyadh, Saudi Arabia; 3 Department of Histopathology, King Saud University, King Khalid University Hospital, Riyadh, Saudi Arabia; University of Newcastle, UNITED KINGDOM

## Abstract

**Background:**

Colorectal cancer is the leading cause of cancer-related deaths in Saudi Arabia. Cancer has a multifactorial nature and can be described as a disease of altered gene expression. The profiling of gene expression has been used to identify cancer subtypes and to predict patients’ responsiveness. Telomere-associated proteins that regulate telomere biology are essential molecules in cancer development. Thus, the present study examined their contributions to colorectal cancer progression in Saudi patients.

**Methods:**

The expression of *hTERT*, *TRF1*, *TRF2*, *POT1*, *ATR*, *ATM*, *Chk1* and *Chk2* were measured via real-time PCR in matched cancerous and adjacent tissues of CRC patients. The protein level of hTERT, TRF1, TRF2, ATR, ATM, Chk1 and Chk2 were measured using immunohistochemistry. A region of *hTERT* core promoter was sequenced via Sanger sequencing. Methylation of CTCF binding site was examined via methylation-specific PCR. Finally, the length of telomere was estimated using q-PCR.

**Results:**

Our results showed that *POT1*, *ATR*, *Chk1 and Chk2* show increased expression in CRC relative to the adjacent mucosa. The expression levels of each gene were associated with clinicopathological characteristics of patients with CRC. There was a positive correlation between the age of the patients and *hTERT* expression. Regarding tumor site, telomere length, *ATR*, *ATM* and *Chk1* were shown to be altered. No somatic mutation was detected in *hTERT* core promoter, and no differences in methylation patterns at CTCF binding site in the promoter between normal and cancer tissues.

**Conclusion:**

Analysis of targeted genes expression in colorectal cancer based on the clinical variables revealed that tumor location and age could have a role in gene expression and telomere length variations and this could be taken under consideration during CRC diagnosis and therapy. Other epigenetic mechanisms could influence *hTERT* expression in cancers. Our findings warrant further validation through experiments involving a larger number of patients.

## Introduction

Colorectal cancer is considered as an important public health issue since it is the third most common malignancy type diagnosed, with more than 1.3 million new cases and 690 thousand deaths worldwide in 2012 [1]. In the Kingdom of Saudi Arabia (KSA), CRC is the leading cause of cancer-related deaths, representing 12% of all cancers and accounting for more than 11% of all cancer cases [1]. In addition, the incidence has increased gradually over the past recent years and this trend is predicted to progress in the future [2, 3].

Around 75% of colorectal cancers are sporadic, whereas 25% could be attributed to family history or inheritance of mutations in genes involved in DNA repair, such as *MLH1* and *MSH2*, or in tumor suppressor genes, such as *APC* [4–7]. The etiological and risk factors of CRC development are variable reflecting the multifactorial nature of the disease. Several risk factors are shown to be associated with CRC development. One of the factors is age, where individual older than 50 years have a higher risk of developing CRC [8]. Another factor contributing to CRC is inflammatory bowel disease [9]. Other risk factors include: personal history of adenomatous polyps, consumption of high-red meat diet, smoking, and the lifestyle with low physical activity [10].

At the molecular level, genetic and epigenetic alterations have been shown to contribute to colorectal carcinogenesis. Telomerase and telomeres are important for chromosomal stability, cell integrity, and multiplication, and multiple studies have provided substantial evidence supporting their role in CRC progression [11, 12]. Human telomeres are special nucleoprotein structures, found at end of linear chromosomes, protecting them from end-to-end fusion and degradation. They also prevent the processing of the DNA terminus as damaged DNA, in need of double-strand break repair [13]. Telomeres consist of hexamer sequence tandem repeats of (TTAGGG)_n_ in the 5'-3' direction, with length ranging from 3 to 20 kb and the length varies among individuals, tissues, and chromosomes [14]. Telomeres are double-stranded and terminate in a single-stranded 3′ G-rich overhang called G-tail. At the terminus, telomeres with the help of several proteins, form a loop named telomere loop (T-loop) that involves the G-tail binding to internal telomeric repeats of the same chromosome, thereby forming a triple-stranded structure called the displacement loop (D-loop) [15, 16].

There are six individual proteins which associated with telomeric DNA, collectively called shelterin complex. They are essential in preventing the recognition of the telomere as single or double strand breaks via the inhibition of Ataxia telangiectasia mutated (ATM) and Ataxia telangiectasia and Rad3 related (ATR) dependent DNA damage response (DDR) pathway and by forming a closed configuration T-loop structure hiding the natural ends that resemble DNA breaks. In addition, they appear to have a role in telomere length homeostasis [17]. The complex is composed of telomeric repeat binding factor 1 and 2 (TRF1, TRF2), which binds the double-stranded repeat regions, and protection of telomeres 1 (POT1), which binds single-stranded G-tail. Three other proteins do not bind directly to DNA but participate through protein-protein interaction. These are telomeric repeat-binding factor 2-interacting protein 1 (RAP1) which binds TRF2, TIN2-interacting protein (TPP1) that binds POT1 and TIN2 which binds three protein simultaneously, TRF1, TRF2 and TPP1 [18]. Accordingly, shelterin proteins afforded a protective mechanism for telomere against homologous recombination (HR) and non-homologous end joining (NHEJ) repair that can lead to different outcomes of increased recombination and deleterious chromosomes fusion events. Other than shelterins, there are a number of proteins that bind to telomere and have a role in T-loop processing such as MRE11, NBS1, RAD51D and others [19, 20].

Telomerase is a nucleoprotein enzyme that has reverse transcriptase activity. One of the main functions of telomerase is maintaining telomere length after each replication cycle [21]. Most cancer cells and stem cells have high telomerase activity, which is required for maintaining cell multiplication. On the other hand, in human normal somatic cells, the activity of telomerase is often undetectable [22].

**Fig 1:**
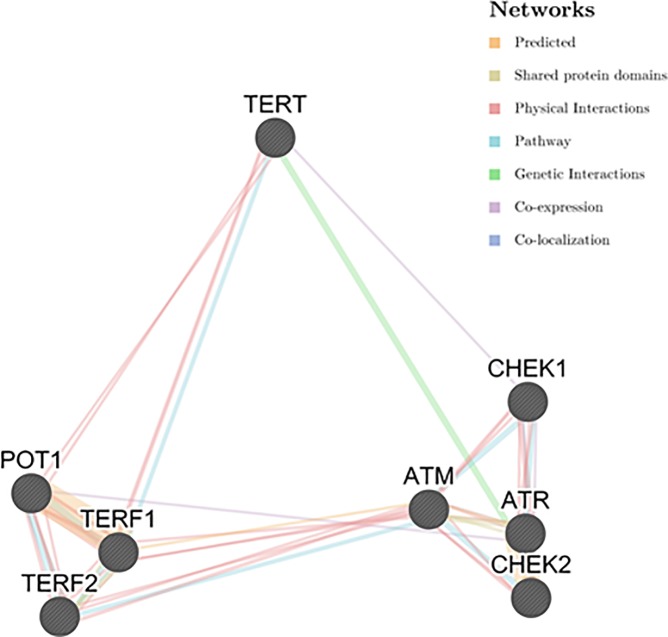
The interaction at different levels between the genes investigated in the present study. hTERT is a subunit of telomerase; TRF1, TRF2 and POT1 are members of sheltering complex; ATM and Chk2 are activated mainly in DNA double-strand breaks (DSBs); ATR and Chk1 can be induced by DNA single-stranded breaks. Source, The GeneMANIA prediction server [23].

In this study, we hypothesized that in CRC the gene expression of the proteins involved in telomere length regulation may be altered. We conducted this study with the aim to understand telomere and the expression of related genes in colorectal cancer progression in Saudi patients. This could help in identifying ‘gene expression signature’. The expression of four telomere-associated proteins, hTERT, TRF1, TRF2, POT1 were studied. In addition to ATM, ATR, Chk1 and Chk2, genes that are involved in DNA damage response and cell cycle checkpoints. Association between the 8 genes studied was assessed used GENEMANIA (Fig 1) [23]. Moreover, in an attempt to investigate the possible mechanisms governed the expression of *hTERT* in CRC tissue, we sequenced the *hTERT* promoter region to identify any somatic mutations that could associate with *hTERT* expression. Furthermore, the methylation status of hTERT was studied. Telomere lengths were measured to identify the effects on CRC, and their associations with *hTERT* and shelterin complex expression. Data were evaluated using matched samples to provide a comprehensive analysis of the genetic and epigenetic properties of cancer-adjacent tissues. This paper presents the first population-based study on the association between telomere-related proteins and CRC.

## Materials and methods

### Patients and samples

The present study was approved by King Saud University ethical review board (IRB) [25 12/3352, College of Medicine]. All procedures were performed after the patient's written informed consent. The study includes a total of fifty (n = 50) Saudi CRC patients who attended to King Khalid University Hospital (KKUH), Riyadh, Saudi Arabia and underwent routine biopsy procedure for their medical treatment and/or diagnosis. They voluntary donated tissues and blood samples for this study. In all cases, colorectal cancer tissue and their adjacent mucosa were obtained prior to treatment and stored in RNAlater solution immediately after excision, to avoid RNA degradation. Blood samples were also collected from of the patients in EDTA containing tube for DNA extraction that was used for sequencing.

Tumor staging was performed according to tumor node metastasis classification (pTNM) system by qualified physicians. Detailed clinicopathological characteristics for each CRC patient including age, gender, tumor site and pTMN stage are presented in Table 1. The median age of the patients was 58 years with a range: 23–79 years. Most of the patients were classified as stage II (46%), and the rest were distributed among other disease stages. Approximately 8% were adenoma (benign) patients.

**Table 1:** Clinicopathological characteristics of CRC patients included in this study.

Characteristic		%
**Age:**	< 57	44
	≥ 57	56
**Gender:**	Male	52
	Female	48
**Tumor site:**	Colon	48
	Rectum	52
**TNM stage:**	Benign	7.7
	Stage I	11.5
	Stage II	46.2
	Stage III	34.6
	Stage IV	0

### Nucleic acid isolation

The extraction of high purity DNA and RNA from colorectal tissues and adjacent mucosa were done using the AllPrep DNA/RNA/Protein Mini Kit (Qiagen). For blood samples, genomic DNA was extracted using the QIAamp DNA Blood Mini Kit (Qiagen). All extractions were performed according to the manufacturer’s instructions. Nucleic acids were quantified and purity was determined spectrophotometrically (NanoDrop 8000, Thermo Fisher Scientific, Epsom, UK).

### Estimation of gene expression via real-time PCR

The preparation of cDNA was carried out prior to real-time PCR (q-PCR) by reverse transcription of purified RNA (2 μg) using high-capacity cDNA Reverse Transcription Kit (Applied Biosystems, USA) according to the manufacturer’s protocol. The expression levels of targeted genes were measured by qPCR (LightCycler 480 II, Roche Applied Science, Switzerland). The levels of hTERT expression were estimated via TaqMan Gene Expression Assay (assay ID Hs00972656_m1; Applied Biosystems, Warrington, UK). The *hTERT* expression was studied in all 50 samples. Due to the shortage of samples and materials, other genes i.e: *TRF1 TRF2*, *POT1*, *ATR*, *ATM*, *Chk1* and *Chk2* were conducted in 20 matched samples using SYBR® Green dye-based assay (Applied Biosystems, Warrington, UK). The expression was normalized using (GAPDH) expression as an endogenous control. The primers sequences used in this study were obtained from previous studies and listed in Table 2. The reaction was carried out in a total volume of 12.5 μl, with 1X power SYBR® Green Master Mix and 0.2 μM of each primer.

**Table 2:** Primers sequences used for the amplification of targeted genes in Q- PCR.

Gene	Primer sequence (5′ - 3′)	*Ta (°C)
GAPDH	Forward: GGT ATC GTG GAA GGA CTC ATG AC	60
	Reverse: ATG CCA GTG AGC TTC CGT TCA GC	
TRF1	Forward: CCACATGATGGAGAAAATTA AGAGTTAT	
	Reverse: TGCCGCTGCCTTCATTAGA	
TRF2	Forward: TCA ATC GCT GGG TGC TCA A	
	Reverse: GTA CCG GCT ACC CCG AAA G	
POT1	Forward: GAA GTG GAC GGA GCA TCA TT	
	Reverse: TTT GTA GCC GAT GGA TGT GA	
ATM	Forward: GCA GAT GAC CAA GAA TGC AA	
	Reverse: GGC CTG CTG TAT GAG CAA AT	
ATR	Forward: GGG ATG CCA CTG CTT GTT ATG AC	
	Reverse: CTG TCC ACT CGG ACC TGT TAG C	
Chk1	Forward: CTTTGGCTTGGCAACAGT	
	Reverse: CCAGTCAGAATACTCCTG	
Chk2	Forward: CTC GGG AGT CGG ATG TTG AG	57
	Reverse: GAG TTT GGC ATC GTG CTG GT	

*Ta: Annealing Temperature

### Validation of gene expression using immunohistochemistry

Immunohistochemistry assay using goat polyclonal Anti-hTERT antibody and Anti-TRF1, Anti-TRF2, Anti-ATR, Anti-ATM, Anti-Chk1 and Anti-Chk2 mouse monoclonal antibodies was performed to compare the difference of the expression among CRC tissues and paired adjacent mucosa using ultraView DAB Detection Kit (Ventana, Arizona, USA) on a BenchMark XT automated staining system (Ventana, Arizona, USA). Briefly, a tissue section of 3-micron thickness was cut using Leica RM2235 Rotary Microtome (Leica Biosystems, Wetzlar, Germany) into a coated slides and allowed to be incubated in a hot air oven at 60°C for 15 min. For routine IHC, tissue sections were then deparaffinized with EZ Prep (Ventana, Arizona, USA) at 75°C. Endogenous peroxidases were blocked with hydrogen peroxide for 4 min. For antigen retrieval tissue sections were pre-treated in Cell Conditioning 1 (CC1; Ventana, Arizona, USA) using standard cell conditioning at 100°C. Tissues were incubated for 32 min at 37°C with Anti-human TERT (1:100, Santa Cruz Biotechnology, USA), TRF1, TRF2, ATR, ATM, Chk1 and Chk2 primary antibodies (1:50, Abcam, USA), then ultraview universal HRP multimer was added as secondary antibody. Visualization of immunolocalized proteins was done by copper-enhanced DAB reaction. Sections were counterstained with Hematoxylin II and Bluing Reagent (Ventana, Arizona, USA) for 4 min. To prevent evaporation and to provide a stable aqueous, liquid coverslip (LCS) was added as a barrier between aqueous reagent and air. Samples were then dehydrated after mounting in DPX by passing through graded alcohol solutions: 70% ethanol, 96% ethanol and absolute ethanol. Then few drops of DPX were added. The reaction buffer was used to wash slides after each step to provide a stable aqueous environment. Immunostained section was reviewed by Olympus BX51 light Microscope and DP72 Olympus Digital Camera with magnification 10X, 20X and 200X (Olympus America Inc, Center Valley, PA, USA) and images were obtained.

### Sequencing of *hTERT* core promoter of blood and tumor tissues

The promoter regions of 24 tumor tissues and their matched blood samples were first amplified (163 bp) using the following set of primers [24]: forward 5'- CAG CGC TGC CTG AAA CTC -3' and reverse 5'- GTC CTG CCC CTT CAC CTT -3' (Invitrogen, USA). Conventional PCR (Biorad T100, USA) was performed using Green Master Mix (Promega, USA). The following PCR profile was used for amplification: initial denaturation, 95°C for 3 min; 34 cycles of 95°C for 30 s, 63°C for 30 s, and 72°C for 30 s; and final extension of 72°C for 15 s. Amplification products were purified using QIAquick Gel Extraction Kit (Qiagen, Germany) according to the manufacturer’s protocol.

The samples were then prepared for Sanger sequencing using Big Dye Terminator 3.1 Cycle Sequencing Kit (Applied Biosystems, USA). Cycle sequencing products were purified using a DyeEx 2.0 Spin Kit (Qiagen). Finally, for sequencing and data analysis, 3500 Genetic Analyzers (Applied Biosystems, USA) was used.

### Methylation-specific PCR (MSP)

DNA methylation pattern in CpG island of CCCTC-binding factor (CTCF) binding region located in *hTERT* was determined by sodium bisulfite treatment, where unmethylated cytosine, but not the methylated, converted to uracil. Around 1 μg of genomic DNA extracted from CRC and normal tissue were converted using EpiTect Bisulfite kit (Qiagen, Hilden, Germany) according to the manufacturer’s instructions. The treated DNA was then subjected to PCR reaction to analyze methylation status, where two specific sets of primers were used, one designed for methylated (M) and the other for un-methylated (U) hTERT promoter. The following primers sequences were used [25]; methylated primers: (forward 5'-GCGCGAGTTTTAGGTAGC-3' and reverse 5'-CTCGCGATAATAACTACGCA-3') and unmethylated primers: (forward 5'-GGTGTGAGTTTTAGGTAGT-3' and reverse 5'-CCTCACAATAATAACTACACA-3'). Parallel with each set of MSP reactions, three controls were used. Those were EpiTect control methylated DNA (Qiagen, Hilden, Germany) as a positive control for the methylated reaction, EpiTect control un-methylated DNA (Qiagen, Hilden, Germany) as a positive control for the unmethylated reaction and water as a negative control. From the treated DNA samples around 500 ng was amplified in 25 μl PCR reaction contained 16.6 mM ammonium sulfate, 67 mM Tris (pH 8.8), 6.7 mM MgCl_2_, 10 mM 2-mercaptoethanol, deoxynucleotide triphosphates (each at 1.25 mM), and 1 U of Platinum Taq DNA polymerase (Invitrogen). Thermocycling was carried out using the following settings: 94°C for 2 min; followed by 35 cycles consisting of 30 s at 94°C, 30 s at 60°C for (M), 56°C for (U), 30 s at 72°C and a final extension at 72°C for 7 min. Amplicons were analyzed by electrophoresis 2% agarose gel, stained with ethidium bromide, and visualized under UV illumination.

### Measurement of relative telomere lengths

Measurement of telomere lengths was conducted using q-PCR on 20 available CRC tissues and their matched normal mucosa. The following two sets of primers were used as previously reported [26]: one for amplification of telomeres (TEL-1 5'-GGTTTTTGAGGGTGAGGGTGAGGGTGAGGGTGAGGGT-3' and TEL-2 5'- TCCCGACTATCCCTATCCCTATCCCTATCCCTATCCCTA-3') and the second set for amplification of the single copy human beta-globin gene (HBG) as (HBG-1 5'-GCTTCTGACACAACTGTGTTCACTAGC-3' and HBG-2 5'-CACCAACTTCATCCACGTTCACC-3').

### Statistical analysis

Relative expression levels and telomere lengths were calculated using the 2^-ΔΔ^Ct method. Software used for statistical analyses and graphs drawing is Graph Pad Prism^®^ program (version 7.03 Graph Pad Software, Inc., San Diego, USA). Statistically significant level was set at p-value < 0.05 for all tests. A nonparametric *t*-test was used to compare any two groups of samples. Person and Spearmen correlation were used for correlation. Sequencing data were assembled using DNA Baser Sequence Assembler V.4.

## Results

### The expression level of *hTERT* in tumor and their adjacent mucosa

**Fig 2:**
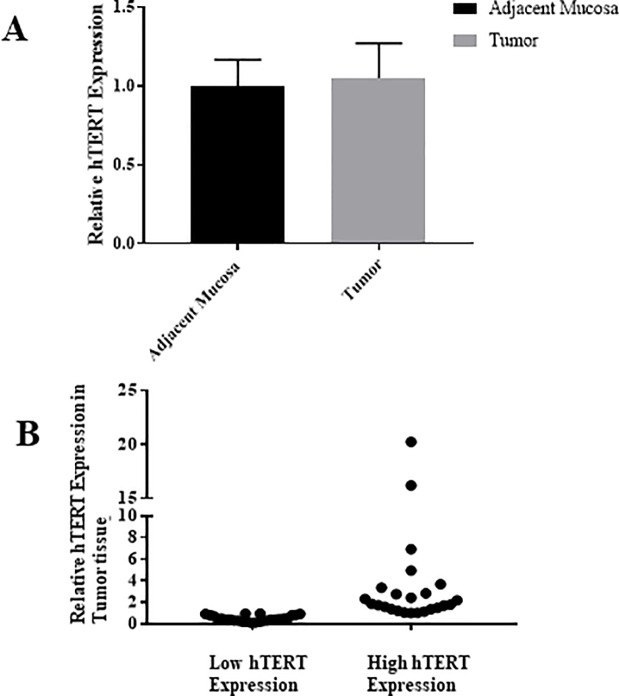
The expression of hTERT in 50 matched CRC tissues relative to the adjacent mucosa. (A) The mean fold change difference between the matched tissues, showing a non-significant increase in CRC tissues relative to the adjacent mucosa (P value > 0.05). (B) The expression patterns of hTERT in each CRC sample relative to its normal mucosa showing a variation among patients, in which some tumor samples showed low hTERT expression, others showed high expression.

All 50 paired samples, tumor tissues and their adjacent cells, showed measurable mRNA levels of *hTERT*. After normalization, no significant difference in *hTERT* expression mean of tumor and adjacent mucosa was observed, as shown in **Fig 2A**. However, after calculating the relative expression of tumor to normal tissue T/N for each paired sample there was a variation between samples (**Fig 2B)**. Thus, CRC patients were divided into two groups based on the relative *hTERT* expression, group I with low *hTERT* expression and group II that showed high expression. The median expression value was used as the cutoff point (= 0.96). The clinicopathological characteristics of each group are listed in **Table 3.**

**Table 3:** Clinicopathological profile of tumors with lower and higher (T/N) *hTERT* expression.

Characteristic		Group I N = 26	Group II N = 24
		%	%
**Age:**	< 60	61.5	45.8
	≥ 60	38.5	54.2
**Gender:**	Male	48.1	58.3
	Female	51.9	41.7
**Tumor site:**	Colon	61.5	41.7
	Rectum	38.5	58.3
**pTNM stage:**	Stage I	6.66	15.38
	Stage II	46.66	38.46
	Stage III	33.33	30.77
	Adenoma	13.33	0

**Fig 3:**
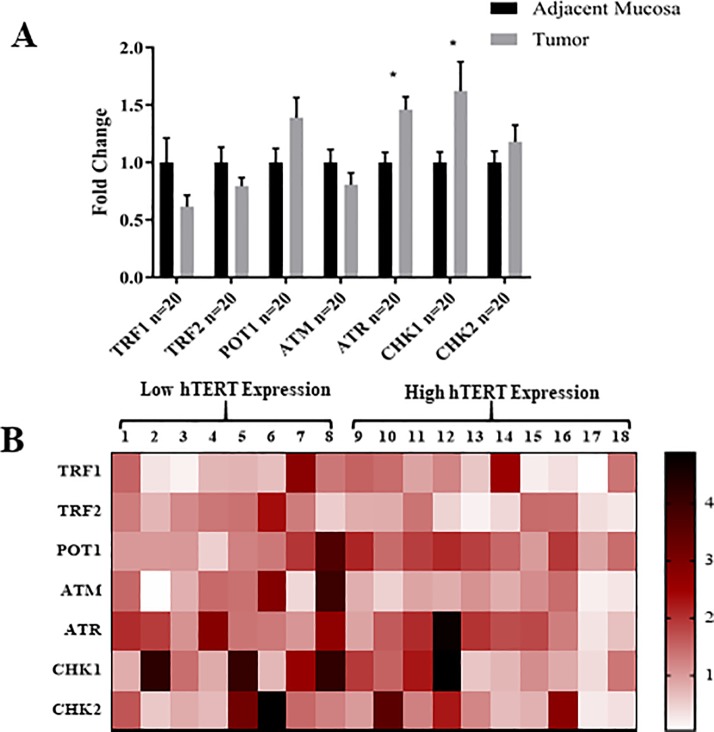
The mean fold change of expression 20 matched tumor and adjacent mucosa. (A) TRF1, TRF2 and ATM showed a non-significant lower expression in tumor, whereas ATR and Chk1 showed significantly higher expression in CRC tissues. The expression of Chk2 and POT1 was higher in tumor tissue compared to the adjacent mucosa, however, the difference was non-significant, * p-value <0.05. (B) The extent of the expression of all genes in 18 patients as Tumor/Normal ratio, showing an approximately same expression extent for each patient.

After that, 20 samples were used in order to examine the expression of *TRF1*, *TRF2*, *POT1*, *ATR*, *ATM*, *Chk1* and *Chk2*. From the results (**Fig 3A)**, it can be seen that *ATR* and *Chk1* are significantly more expressed in CRC tissues compared to their adjacent mucosa. On the contrary, *TRF1*, *TRF2* and *ATM* showed lower expression in tumor, statistically, however, the difference was not significant. In addition, *Chk2* showed a nonsignificant increased expression in the tumor relative to the adjacent mucosa.

To obtain a better understanding of the mechanisms and to have an overview about how these genes are correlated to each other in these samples, the fold change in tumor of each gene is presented in each samples (**Fig 3B).**

Correlation study revealed that samples which had a higher expression of *hTERT*, tend to have a lower expression of *TFR1*, *TRF2* and the genes related to DNA damage response, however, the correlation was not statistically significant. On other hand, a significant positive correlation was found between each of *TRF1* and *POT1*, *TRF2* and *ATM*, *TRF2* and *Chk2*, *ATM* and *ATR*, *ATM* and *Chk2* **Table 4**.

**Table 4:** Spearman correlation coefficient between the expressions of examined genes.

	TRF1	TRF2	POT1	ATM	ATR	Chk1	Chk2
hTERT	-0.075	-0.329	0.218	-0.230	-0.298	-0.294	-0.265
TRF1		-0.156	*0.550	0.011	0.051	0.243	0.240
TRF2			-0.183	*0.494	0.046	0.051	*0.515
POT1				0.098	0.057	0.424	0.399
ATM					*0.513	-0.164	*0.519
ATR						0.255	0.183
Chk1							0.207

*p-value is less than 0.05

### Effect of tumor characteristics on gene expression

**Fig 4:**
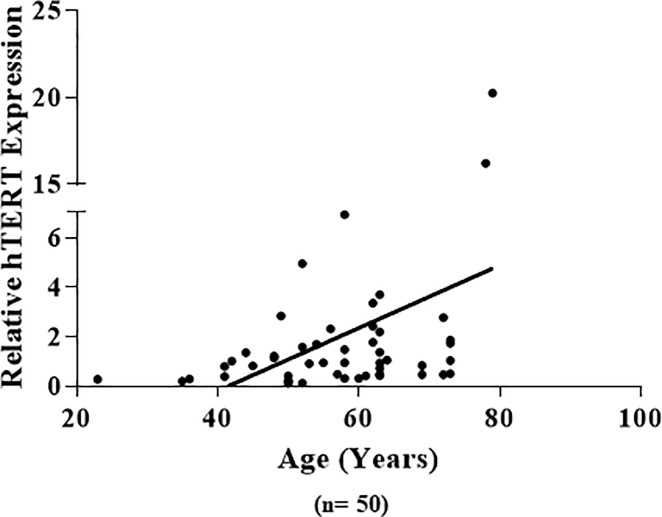
A correlative plot of relative hTERT expression and patient's age. Positive correlation is observed, Correlation coefficient = 0.411, p-value <0.01.

The expression of each gene was correlated to the gender, age of onset, tumor location (colon and rectum) and pTNM stage of cancer. **Table 5** summarizes the relationship between clinicopathological characteristics of tumor and the expression of the 8 investigated genes. Main findings include the following: *hTERT* expression showed a positive correlation with age of the patients, in which patients who are 57 years or younger showed lower expression compared to patients who are more than 57 years old (**Fig 4)**. The expression of *ATR* appeared to be higher in the tumor of patients who are over 57 years of age compared to adjacent mucosa (p-value = 0.02). The difference in expression of *Chk2* between the two age groups was nearly significant (p-value = 0.052) where younger patients showed higher expression. The effect of gender on the expression of the studied genes revealed that for *ATR* females had two-fold higher expression compared to males, whereas *POT1* was expressed at a significantly higher level in the tumor tissue relative to adjacent mucosa only in the male patients. None of the other genes showed any difference between males and females. Regarding tumor site, the DNA damage response genes, *ATR*, *ATM* and *Chk1*, showed a difference in their expression. Tumor located in colon had higher expression of *ATR* than its adjacent mucosa. On the contrary, rectum shows a significant higher *Chk1* expression in the tumor tissue. Furthermore, expression of *ATM* was significantly lower in rectum tumor compared to its adjacent tissue, whereas almost no change in its expression between colon cancer and its adjacent mucosa. The patients were grouped based on tumor stage, stage I & II (early stage), stage III & IV (late stage). The only gene appeared to be affected by stage was ATR, a significant increase in expression of tumor tissue of the late stage was seen compared to its adjacent mucosa.

**Table 5:** Mean relative expression of candidate genes with clinicopathological variables.

	Characteristic	Age		Gender		Tumor site		TNM stage	
		≤ 57	> 57	Male	Female	Colon	Rectum	I&II	III&IV
	% for 50 samples	44	56	52	48	48	52	57.7	34.6
	% for 20 samples	45	55	45	55	55	45	35	65
**TERT**°	Mean ±SEM	0.67 ±0.12	1.47 ±0.38	1.30 ±0.38	0.86±0.25	0.96± 0.23	1.4 ± 0.43	1.5±0.2	0.8±0.2
	P-value	0.05*		n.s		n.s		n.s	
**TRF1**	Mean ±SEM	0.77 ±0.13	0.53 ±0.14	0.47 ±0.16	0.82 ±0.11	0.68 ± 0.09	0.57 ±0.16	0.79 ±0.25	0.52 ±0.07
	P-value	n.s		n.s		n.s		n.s	
**TRF2**	Mean ±SEM	0.80±0.15	0.79±0.08	0.72±0.1	0.88±0.12	0.88±0.12	0.71±0.09	0.66±0.12	0.87±0.09
	P-value	n.s		n.s		n.s		n.s	
**POT1**	Mean ±SEM	1.46 ±0.31	1.34 ±0.20	*1.47 ±0.14	1.31 ±0.31	1.30 ±0.25	1.46 ±0.18	1.40 ±0.44	1.39 ±0.16
	P-value	n.s		n.s		n.s		n.s	
**ATR**	Mean ±SEM	1.33 ±0.14	*1.59 ±0.18	1.08 ±0.14	**2.04 ±0.20	**1.66 ± 0.17	1.28 ±0.16	1.5 ±0.19	*1.45 ±0.14
	P-value	n.s		0.009**		n.s		n.s	
**ATM**	Mean ±SEM	0.81±0.17	0.81±0.12	0.67±0.14	0.93±0.15	1.09±0.18	*0.58±0.12	0.78±0.20	0.83±0.12
	P-value	n.s		n.s		0.029*		n.s	
**Chk1**	Mean ±SEM	1.06 ±0.11	1.37 ±0.14	2.08 ±0.50	1.38 ±0.31	1.31 ± 0.33	*2.16 ±0.42	1.98 ±0.50	1.38 ±0.24
	P-value	n.s		n.s		n.s		n.s	
**Chk2**	Mean ±SEM	1.40±0.11	0.90±0.21	1.30±0.29	1.09±0.12	0.96±0.08	1.54±0.35	1.26±0.35	1.14±0.13
	P-value	n.s		n.s		n.s		n.s	

°hTERT clinical data analysis based on 50 samples, others gene on 20 samples of the 50.

* (pvalue < 0.05)

** (pvalue < 0.01)

### Immunohistochemistry

**Fig 5:**
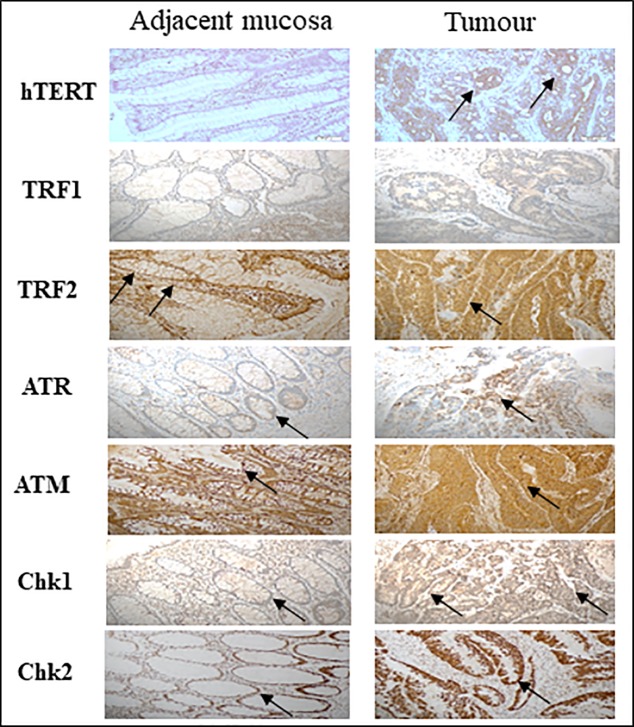
Immunohistochemical staining for TRF1, TRF2, ATR, ATM, Chk1 and Chk2 in paraffin-embedded colorectal carcinoma and adjacent mucosa. Abundant expression of hTERT, TRF2, ATR, ATM and Chk2 were observed in CRC tissue compared to adjacent mucosa validating the q-PCR results. No difference was detected in TRF1 protein level between the cancerous tissue and adjacent mucosa.

To further validate the q-PCR results, immunohistochemical staining using anti-TRF1, anti-TRF2, anti-ATM, anti-ATR, anti-Chk1, anti-Chk2 and anti-TERT antibodies were carried out. Representative results of TRF1, TRF2, ATM, ATR, Chk1, Chk2 and TERT immunostaining of colorectal cancer tissue and paired adjacent mucosa are presented in **Fig 5**. Samples were chosen randomly and then were compared to q-PCR results. A strong staining was detected in colorectal carcinoma tissue relative to matched normal tissue for all the genes, that were consistent with the q-PCR results, except for TRF1 which shows no noticeable difference in staining between tumor and adjacent tissues. This was in disagreement with the result of q-PCR.

Staining for TRF2 was localized in nucleus and cytoplasm, while ATM showed mainly a cytoplasmic stain. On the other hand, ATR, Chk1 and Chk2 showed a clear nuclear staining. hTERT expression was found to be heterogeneous among observed subjects. Overall hTERT showed strong-moderate staining in CRC tissues and was localized in nucleus and cytoplasm, while negative-week staining was observed in adjacent tissues.

### Sequencing of the *hTERT* promoter region

**Fig 6:**
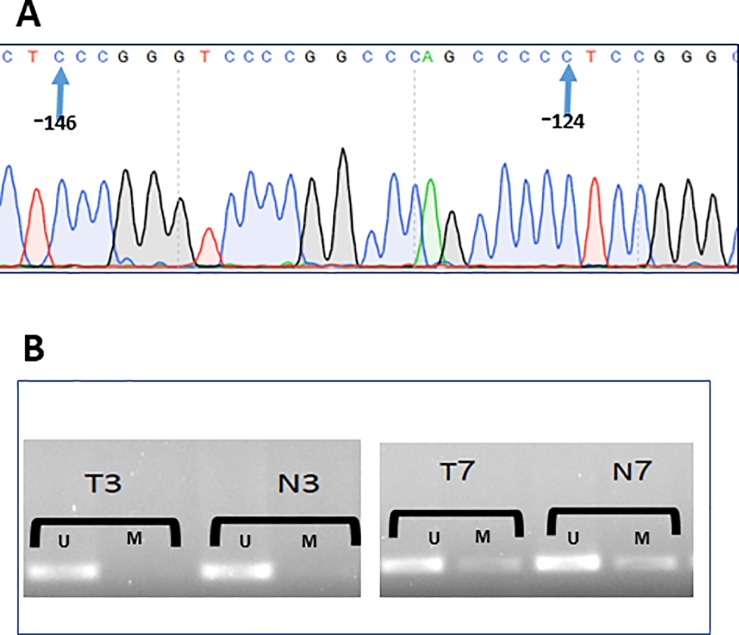
Promoter sequencing and methylation status of hTERT. (A) Chromatogram of a part of hTERT core promoter region, no mutation was detected in any of the samples (n = 24); (B). Majority of samples showed unmethylation n = 13 (Left), some are partially methylated n = 3 (Right), U: Unmethylated; M: Methylated, T: Tumor; N: Adjacent mucosa.

The promoter region for hTERT was sequenced in 24 cancer tissues and matched blood samples. Sequence obtained for each patient matched those of the wild-type samples. No mutation was detected in the area examined (**Fig 6A).**

### Methylation of the CTCF-binding region of the *hTERT* promoter

MSP study was carried out for 16 samples to investigate whether the alteration of the methylation pattern of CTCF binding region of *hTERT* in colorectal cancer correlated with the aberrant *hTERT* expression pattern. The results demonstrated no difference in the methylation status in tumor tissue and the adjacent mucosa. Majority of the samples (13 pairs) showed unmethylated region, the remaining appeared to be partially methylated. Representative samples of gel analysis of MSP are presented in **Fig 6B**.

### Relative telomere lengths in CRC tissues to normal mucosa

**Fig 7:**
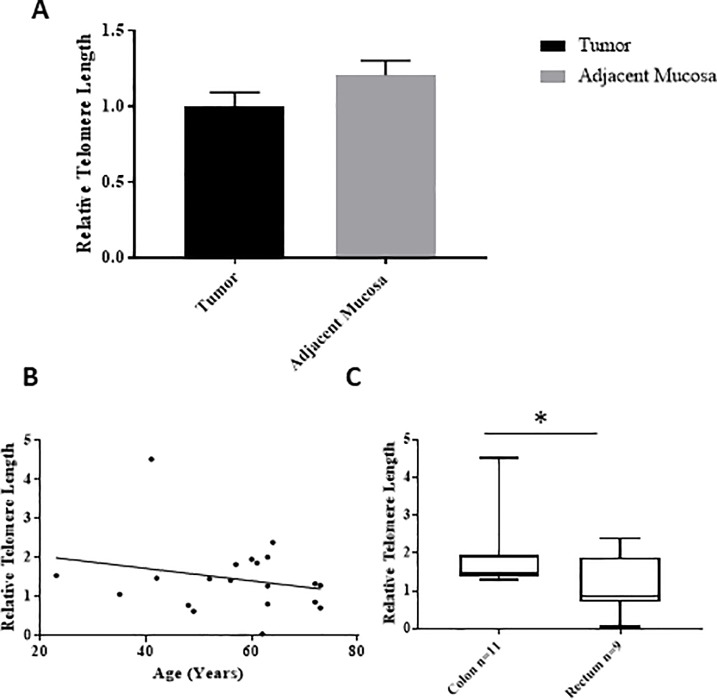
Telomere length analysis. **A**. A comparison of telomere length of tumor tissue and adjacent mucosa (n = 20), there was no significant difference (p-value > 0.05), **B**. Correlation between age and telomere length showed non-significant weak negative correlation with telomere length, Spearman correlation r -0.175, p-value >0.05. **C**. Comparison of telomere length in the tumor of the colon (n = 11) and rectum (n = 9). In the colon, the telomere length was significantly higher compared to the rectum (p <0.05).

Relative telomere lengths in 20 selected matched samples were determined via q-PCR, with beta-globin as the single copy gene (SCG). Telomere length was calculated as the relative ratio between the telomere and SCG (Telomere/SCG or T/S). Upon analysis, there was no difference in telomere length between cancer tissue and adjacent mucosa (**Fig 7A).**

The tumor samples were grouped according to the age at diagnosis and relative telomere length was correlated with age, showing a negative correlation (r value = -0.175, p>0.05) (**Fig 7B)**; however, the association was not statically significant. Furthermore, the samples were grouped according to the tumor location (i.e colon or rectum) and the results are presented in **Fig 7C.** Relative telomere length was higher in the tumor of colon compared to the tumor located in rectum (p<0.05).

The rate of expression of the eight genes was correlated with the telomere length in the 20 studied samples. The expression of *hTERT* found to be the only gene to be correlated with telomere length. Other genes did not show any correlation **Table 6.**

**Table 6:** Correlation between expression of eight studied genes and telomere length.

Gene	Correlation with Telomere Length	P value
**hTERT**	-0.643	*
**TRF1**	0.069	n.s
**TRF2**	-0.063	n.s
**POT1**	-0.102	n.s
**ATM**	-0.044	n.s
**ATR**	0.207	n.s
**CHK1**	0.191	n.s
**CHK2**	-0.001	n.s

*correspond to a significant value

## Discussion

Studying molecular alterations that involve in cancer initiation and/or progression is one of the most important aspects to understand cancer biology. Colorectal cancer is a multifaceted disease; thus, CRC initiation and/or progression is unlikely to be caused by a single gene or enzyme. Nevertheless, illustrating the role of any given molecule in CRC is an important task that can contribute to deciphering the complexity of CRC. Finding a ‘gene expression signature’ of a single or combined gene expression alteration could help in diagnosis, prognosis, understanding the reasons behind drug resistance and prediction of therapeutic response.

The estimation of gene expression and telomere length in CRC tissues relative to their adjacent mucosa was performed using q-PCR. All samples expressed *hTERT*, however, there was no difference in the mean between tumor and adjacent mucosa. Tissues adjacent to tumors were macroscopically normal but showed detectable *hTERT* mRNA levels. Many studies have shown that normal tissues do not express *hTERT* [27]. Thus, our findings can be attributed to the presence of cancer-associated genetic modifications [28]. A previous study identified a number of genes that are activated in the adjacent mucosa of CRC patients, and the same genes were found to be repressed in the mucosa of healthy donors [29]. In addition, others demonstrated that telomerase activities in mucosa located ~5 cm away from the tumor were higher than those in mucosa collected 10 cm from the tumor [30]. Thus, *hTERT* expression in the adjacent mucosa can vary based on proximity to the primary tumor and can be triggered by factors either released from the tumor or in response to the tumor [31, 32]. Furthermore, correlation study revealed a negative correlation between relative *hTERT* expression and relative telomere length, unlike other studied genes where no significant correlation was found. This negative correlation is consistent with the concept that telomere length is associated with the initial kinetics of telomere shortening, instead of the association with *hTERT* expression [32].

In addition, cancer cells are frequently associated with aberrant cell cycle progression, checkpoints dysfunction, impaired DNA repair, genomic instability and/or defective apoptosis pathways. Our results showed that expression of *ATR* and *Chk1* were significantly increased in cancer tissues, as show the mRNA and protein levels. The ATR-Chk1 pathway is triggered by RPA-coated single-stranded DNA, and it is known that this is crucial in preventing genomic DNA damage. Thus, *ATR* and *Chk1* appear to be even more important in tumor than normal cells, in which it is critical for cancer cells for survival, in coping replication stress and in preventing cell death. Moreover, ATR expression was correlated with ATM which also involved in DNA damage response gene, which magnifies the crosstalk between pathways [33]. A positive correlation was found between ATM and Chk2, which is a key downstream target of ATM [34]. A study found that the upregulation of these genes leads to poor prognosis [35]. Indeed, the expression of ATR/Chk1 and ATM/Chk2 pathways and thus related responses and repair pathways can serve as a biomarker of response tendency to certain therapies that induce such types of DNA damage.

Analysis of targeted genes expression in colorectal cancer based on the clinical and pathological variables revealed that tumor location could have an important role in gene expression variation. The expression of ATM in tumors located in rectum was lower compared to its adjacent mucosa, in contrast to ATR, which showed a significant increased expression in colon cancer relative to its adjacent mucosa. Chk1 showed a two-fold increase in expression in rectum compared to the matched mucosa. The ATM gene is mainly activated in cases of DSBs, while unlike ATM, ATR is activated by a broad spectrum of DNA damages in addition to DSBs [36]. The difference in ATR, ATM and Chk1 expression pattern between rectal and colon cancer strength that of considering colon and rectum are distinctive organs rather than one common entity, at least at the molecular level. In addition, tumor location showed a difference in the Chk2 expression, where rectum cancer tissues had a higher Chk2 expression in compared to normal, while colon cancer tissue showed a slight decrease in its expression. Although the difference between the rectal and colon cancer was nonsignificant, the difference in the Chk2 expression pattern suggests that tumor location is an important factor affecting Chk2 expression.

Supporting this opinion, our results found that telomere length was shorter in rectum cancer relative to colon cancer. This observed variation in expression among tumor locations could arise from the fact that they originate from different cells during embryo development. Moreover, the arteries that supply blood to these locations are different [37]. Main additional differences include: gradient of hormone receptors, in which they are less concentrated in the proximal colon and more concentrated in the rectum [38, 39]; the mutation rates in rectal cancer are higher[40]; and the molecular mechanism of tumor development is not the same based on the location, CIN incidence is high in rectal cancer and MSI in proximal colon cancer [41, 42]. Some previous studies showed differences in expression between CIN and MSI [43]. However, other studies did not find significant differences [44].

Interestingly, none of the expression of the studied genes was altered by the age of the patients except *ATR* and *hTERT*. The positive correlation between age and relative *hTERT* expression indicates that *hTERT* expression could be influenced by age; other studies have also reported similar pattern [45].

In agreement with previous studies, there was no significant association between gender and the expression of hTERT [46]. However, in this study, the gender of the patients is shown that it could affect ATR expression, which showed 100% more expression in females than males, whereas its shelterin member inhibitor at telomere, POT1, was significantly increased in CRC male patients relative to their adjacent mucosa.

To investigate the possible mechanisms governed the expression of *hTERT* in CRC tissue, we investigated any mutation in the promoter region and the role played by methylation on gene expression. Sequencing of the *hTERT* promoter regions did not reveal any mutations. These findings are consistent with the results of other studies, which showed that mutations found at locations -146 and -124 of the *hTERT* promoter region were tissue-specific and were most common in tissues with relatively low rates of self-renewal [47]. Therefore, other mechanisms could regulate *hTERT* expression.

The examination of the methylation status of CTCF binding site in *hTERT* promoter by MSP results in a similar methylation pattern between tumor samples and those of adjacent tissues regardless of *hTERT* expression levels. A previous study has shown that methylation of the CTCF binding site could upregulate *hTERT* expression, and it was explained that methylation in this area prevents the binding of the CTCF repressor, additionally, several studies confirmed the hypermethylation of *hTERT* in various adenocarcinoma cell line models [25, 48]. Depending on the results, alteration of the methylation status of CTCF binding site in *hTERT* promoter seems to be not a potential mechanism for its transcriptional regulation in colorectal cancer. Indeed, other mechanisms may contribute to its expression regulation like microRNAs. In fact, other studies showed the significant role played by microRNA in hTERT regulation, such as miR-138 [49].

In summary, the results of this study could participate in improving the understanding CRC among Saudi patients. Analysis of targeted genes expression in colorectal cancer based on the clinical and pathological variables revealed that tumor location and age could have an important role in gene expression and telomere length variations and this could be taken under consideration during CRC diagnosis and therapy, particularly those drugs that based on DNA damage-inducing or targeting. In addition, CRC should not be considered as a single disease at the molecular level. The promoter region of hTERT in CRC samples examined did not harbor any mutations. In addition, there were no differences in the methylation of the CTCF-binding sites, suggesting that other epigenetics mechanisms, such as methylation in other regions of promoter or miRNAs, influence *hTERT* expression in CRC. Our findings warrant further validation through experiments involving a larger number of patients.
